# Breast Development and Cancer

**DOI:** 10.3390/cancers15061731

**Published:** 2023-03-13

**Authors:** Martine Berliere, Francois P. Duhoux, Aline François, Christine Galant

**Affiliations:** 1 Breast Clinic, King Albert II Cancer Institute, Cliniques Universitaires Saint-Luc, Avenue Hippocrate, 10, 1200 Brussels, Belgium; 2 Department of Gynecology, King Albert II Cancer Institute, Cliniques Universitaires Saint-Luc, Avenue Hippocrate, 10, 1200 Brussels, Belgium; 3 Department of Medical Oncology, King Albert II Cancer Institute, Cliniques Universitaires Saint-Luc, Avenue Hippocrate, 10, 1200 Brussels, Belgium; 4 Department of Pathology, King Albert II, Cancer Institute, Cliniques Universitaires Saint-Luc, Avenue Hippocrate, 10, 1200 Brussels, Belgium

The human breast, as mentioned by Gudjonsson and co-authors [1], is a unique, original and dynamic organ, as most of its development occurs postnatally between menarche and menopause. This development comprises different phases of proliferation, differentiation and apoptosis. Highly particular phases occur during pregnancy and lactation [1,2,3]. The steps of breast development may be compared to a sophisticated choreography highlighting the interactions between different types of cells (stem cells, epithelial and stromal cells), hormones (estrogens, progesterone and prolactin), autocrine and paracrine factors [1,2]. Stem cells and cancer cells share several similarities, such as increased survival and cellular plasticity [1]. Understanding the extraordinary development of the normal mammary gland is key to elucidating the genesis of breast cancer. The cellular origin of breast cancer has been debated for decades, but accumulating evidence is linked to mutations in stem or progenitor cells within the epithelial compartment, and these mutations can give rise to the various histopathological subtypes of breast cancer. Among the breast cancer subtypes, triple-negative breast cancer (TNBC) is a devastating disease which represents less than 20% of all breast cancer subtypes but is responsible for the most breast-cancer-related deaths [4]. The study of Rana and co-authors shows, for the first time, that *YB1*, a multifunction gene, plays a major role in shaping the TNBC disparities observed between African American and Caucasian American women. Their data strongly support the notion of an interplay between *YB1* and chemoresistance in driving the aggressivity of TNBC tumors in African Americans.

Further expanding the landscape of mutations observed in breast cancers, Shen et al. present a study of mutations of the Hairless gene (*HR*), which encodes a transcription factor with histone demethylase activity that is essential for the development of tissue homeostasis. The mutational inactivation of *HR* promotes tumorigenesis [5]. The authors identified *HR* as a novel tumor suppressor gene that is frequently mutated in breast cancer.

The steps following breast cancer initiation are cancer progression and metastasis. In this context, Wang and co-authors established that Kindlin-2, within the mammary gland microenvironment, is a major driver of tumor progression and metastasis [6].

The main objective of the molecular decoding of cancers is to develop innovative therapeutic strategies. In this regard, the work of Aqil et al. shows that the oral administration of bilberry-derived anthocyanidins (Anthos) can inhibit the growth and metastasis of TNBC and chemosensitize paclitaxel (PAC)-resistant TNBC cells by modulating the NF-kB signaling pathway, as well as metastatic and angiogenic mediators [7]. This approach provides a highly promising and effective strategy for the management of TNBC.

With the same objective of developing innovative and alternative strategies for another molecular subtype of breast cancer, Krutilima et al. demonstrate that sabizabulin, a potent, orally bioavailable colchicine binding site agent, suppresses HER2 breast cancer and metastasis. The authors show that sabizabulin is a promising alternative agent that can be used to target tubulin in HER2 breast cancer, showing a similar anti-metastatic efficacy to paclitaxel but with the advantages of oral bioavailability and a lower toxicity than taxanes [8].

When breast cancer is present, its detection in the early stages is associated with improved survival, meaning that fewer intensive treatments are required. The current method of breast cancer screening via mammography is unsuitable for use among (younger) women with more dense breasts and also has limitations in its ability to detect aggressive breast cancers. Crook and colleagues describe a breast cancer detection test that is based on the detection of circulating tumor cells in blood samples [9]. This test can detect CTCs with high accuracy across all age groups, hormone receptor subtypes, histological subtypes and disease grades. The results show that this test has a negligible risk of false positive findings, as well as a high detection rate for early-stage (localized) breast cancer. This promising test can thus improve the accuracy of breast cancer detection and could also improve and facilitate the screening of transgender women [2].

After the detection of breast cancer, treatment takes place. New concepts also include surgery and subsequent treatments.

In this area, conservative approaches have radically changed the concept of healing, focusing also on the psychological aspect of oncological treatments. In this scenario, radiotherapy plays a key role. The review written by Cozzi extensively describes the indications, applications and advantages of interstitial brachytherapy [10].

In conclusion, this Special Issue shows that continued progress is being made in our understanding of normal breast development and breast cancer development. Based on molecular discoveries, exciting new therapeutic perspectives are arising. The future looks bright and exciting.
